# Differential miRNA expression profiles in the bone marrow of Beagle dogs at different stages of *Toxocara canis* infection

**DOI:** 10.1186/s12864-022-09081-8

**Published:** 2022-12-22

**Authors:** Jin Gao, Yang Zou, Xiao-Jing Wu, Yue Xu, Xing-Quan Zhu, Wen-Bin Zheng

**Affiliations:** 1 Laboratory of Parasitic Diseases, College of Veterinary Medicine, Shanxi Agricultural University, Taigu, 030801 Shanxi Province China; 2 State Key Laboratory of Veterinary Etiological Biology, Key Laboratory of Veterinary Parasitology of Gansu Province, Lanzhou Veterinary Research Institute, Chinese Academy of Agricultural Sciences, Lanzhou, 730046 Gansu Province China; 3 Key Laboratory of Veterinary Public Health of Higher Education of Yunnan Province, College of Veterinary Medicine, Yunnan Agricultural University, Kunming, 650201 Yunnan Province China

**Keywords:** *Toxocara canis*, Toxocariasis, Beagle dog, Bone marrow, miRNAs

## Abstract

**Background:**

*Toxocara canis* is distributed worldwide, posing a serious threat to both human and dog health; however, the pathogenesis of *T. canis* infection in dogs remains unclear. In this study, the changes in microRNA (miRNA) expression profiles in the bone marrow of Beagle dogs were investigated by RNA-seq and bioinformatics analysis.

**Results:**

Thirty-nine differentially expressed (DE) miRNAs (DEmiRNAs) were identified in this study. Among these, four DEmiRNAs were identified at 24 h post-infection (hpi) and all were up-regulated; eight DEmiRNAs were identified with two up-regulated miRNAs and six down-regulated miRNAs at 96 hpi; 27 DEmiRNAs were identified with 13 up-regulated miRNAs and 14 down-regulated miRNAs at 36 days post-infection (dpi). Among these DEmiRNAs, cfa-miR-193b participates in the immune response by regulating the target gene *cd22* at 24 hpi. The novel_328 could participate in the inflammatory and immune responses through regulating the target genes *tgfb1* and *tespa1*, enhancing the immune response of the host and inhibiting the infection of *T. canis* at 96 hpi. In addition, cfa-miR-331 and novel_129 were associated with immune response and self-protection mechanisms at 36 dpi. 20 pathways were significantly enriched by KEGG pathway analysis, most of which were related to inflammatory response, immune response and cell differentiation, such as Cell adhesion molecules (CAMs), ECM-receptor interaction and Focal adhesion.

**Conclusions:**

These findings suggested that miRNAs of Beagle dog bone marrow play important roles in the pathogenesis of *T. canis* infection in dogs and provided useful resources to better understand the interaction between *T. canis* and the hosts.

**Supplementary Information:**

The online version contains supplementary material available at 10.1186/s12864-022-09081-8.

## Background

*Toxocara canis* is one of the most common causative agents of toxocariasis that is a neglected zoonosis with a global distribution, causing ocular larva migrans (OLM), visceral larva migrans (VLM) or neurotoxocariasis (NT) in humans and animals [1]. The global prevalence of *Toxocara* infection in humans equals 19%, extrapolating to the global population, approximately 1.4 billion people worldwide were estimated to be infected with or threatened by *Toxocara*, with 8%, 11%, 13%, 24%, 28%, 34% and 38% in the Eastern Mediterranean region, the Europe, the North Americas, the Western Pacific regions, the South Americas, the South-East Asia and African, respectively [2]. Multiple factors may affect the prevalence of *Toxocara* infection in different regions, such as socio-ecological, environmental and genetic effects [3]. The global prevalence of *T. canis* infection in dogs equals 11.1%, and dogs infected with *T. canis* can continuously excrete eggs into the environment, contaminating water sources and vegetation [4]. When dogs are infected with infectious eggs of *T. canis*, the larvae can be hatched in the duodenum within 2–4 h. Then the larvae penetrate the intestinal wall and migrate to reach the liver at approximately 24 hpi; subsequently, the larvae continue migration with blood flow, passing the heart and reaching the lung at 96 hpi; the larvae then return to the digestive tract through the trachea and throat; after 36 dpi, the larvae develop into adults in the intestinal tract [5]. *T. canis* has a complex life cycle in definitive hosts and paratenic hosts, which ensures that *T. canis* persists in hosts [5].

Bone marrow, an important hematopoietic and immune organ in the body, can produce red blood cells (RBCs), leukomonocytes and various white blood cells, which play indispensable roles in the host’s defense against parasite infection [6]. For example, bone marrow-derived eosinophils with cytotoxic functions can damage the worms that invade the host [7]. Genomics, transcriptomics, proteomics and metabolomics are widely used to explore the biological functions of genes, proteins and small metabolites of *T. canis* as well as the *T. canis*-host interactions [8–11].

miRNAs are a class of short, single-stranded, non-coding RNAs of about 22 nucleotides (nt) in length and can affect the gene expression post-transcriptionally [12]. miRNAs not only can regulate the development, differentiation and apoptosis in cells, but also participate in the development process of many diseases [13]. Over the past decade, thousands of miRNAs have been identified in helminths, many of them were associated with parasite development and interactions of parasites-hosts [14]. For example, some miRNAs of *T. canis* were predicted to be associated with the development, reproduction, host-parasite interactions or drug resistance [15]. Altered host miRNAs induced by *T. canis* infection play important roles in the pathogenesis of parasite migration in the liver [16]. Competing endogenous RNA (ceRNA) network analysis of lncRNA-miRNA-mRNA in puppy lung after *T. canis* infection improved the understanding of the miRNA regulatory mechanisms [17]. However, the effect of *T. canis* infection on the expression of miRNAs in dog bone marrow remains unclear.

In this study, the expression trajectories of miRNAs in the bone marrow of Beagle dogs were profiled by RNA-seq technology at different time points after *T. canis* infection. Through the bioinformatics analysis, we identified several differentially expressed (DE) miRNAs (DEmiRNAs) that could play regulatory roles in immune response in infected dogs, which may contribute to understanding the pathogenesis of toxocariasis.

## Results

### Characterization of the RNA sequencing data

A total of 272,834,730 raw reads and 264,942,218 clean reads were obtained by RNA-seq. The average raw data of each sample was 0.75 Gb. A total of 585 miRNAs were identified in this study, including 321 known miRNAs and 264 novel miRNAs. The Q20 and Q30 of the raw data were both > 95%, and the percentage of GC was approximately 50% (Table 1), suggesting that these RNA-seq data were of good quality and can be used for subsequent bioinformatics analysis.

**Table 1:** Quality control results of sequencing data

Group	Sample	Total reads	Clean reads	Bases	Error rate	Q20	Q30	GC content
24 hpi	BM24hT1	13,799,657	13,426,802	0.690G	0.00%	99.85%	99.58%	49.17%
	BM24hT2	14,941,957	14,168,878	0.747G	0.01%	99.74%	99.30%	49.27%
	BM24hT3	15,314,418	14,884,866	0.766G	0.00%	99.85%	99.58%	49.74%
	BM24hC1	20,323,844	19,639,690	1.016G	0.01%	99.75%	99.30%	49.01%
	BM24hC2	12,442,986	12,108,245	0.622G	0.00%	99.83%	99.54%	49.11%
	BM24hC3	15,238,083	14,861,267	0.762G	0.00%	99.82%	99.48%	49.06%
96 hpi	CM96hT1	18,730,690	18,196,734	0.937G	0.01%	99.65%	99.08%	49.15%
	CM96hT2	17,725,623	17,224,265	0.886G	0.01%	99.75%	99.34%	48.92%
	CM96hT3	10,691,329	10,376,809	0.535G	0.00%	99.86%	99.61%	49.60%
	CM96hC1	16,028,228	15,694,523	0.801G	0.01%	99.71%	99.28%	49.47%
	CM96hC2	15,644,646	15,229,638	0.782G	0.00%	99.88%	99.64%	48.99%
	CM96hC3	13,380,695	13,063,955	0.669G	0.00%	99.82%	99.51%	49.02%
36 dpi	DM36dT1	10,596,623	10,347,631	0.530G	0.00%	99.86%	99.60%	49.23%
	DM36dT2	18,457,852	17,889,201	0.923G	0.01%	99.58%	98.91%	49.07%
	DM36dT3	15,081,507	14,645,379	0.754G	0.01%	99.51%	98.85%	49.57%
	DM36dC1	14,649,980	14,313,896	0.732G	0.01%	99.74%	99.35%	48.83%
	DM36dC2	15,081,270	14,485,680	0.754G	0.01%	99.72%	99.30%	49.01%
	DM36dC3	14,705,342	14,384,759	0.735G	0.01%	99.76%	99.34%	49.20%

### Differential expression of miRNAs and qRT-PCR validation of the RNA-seq results

**Fig. 1:**
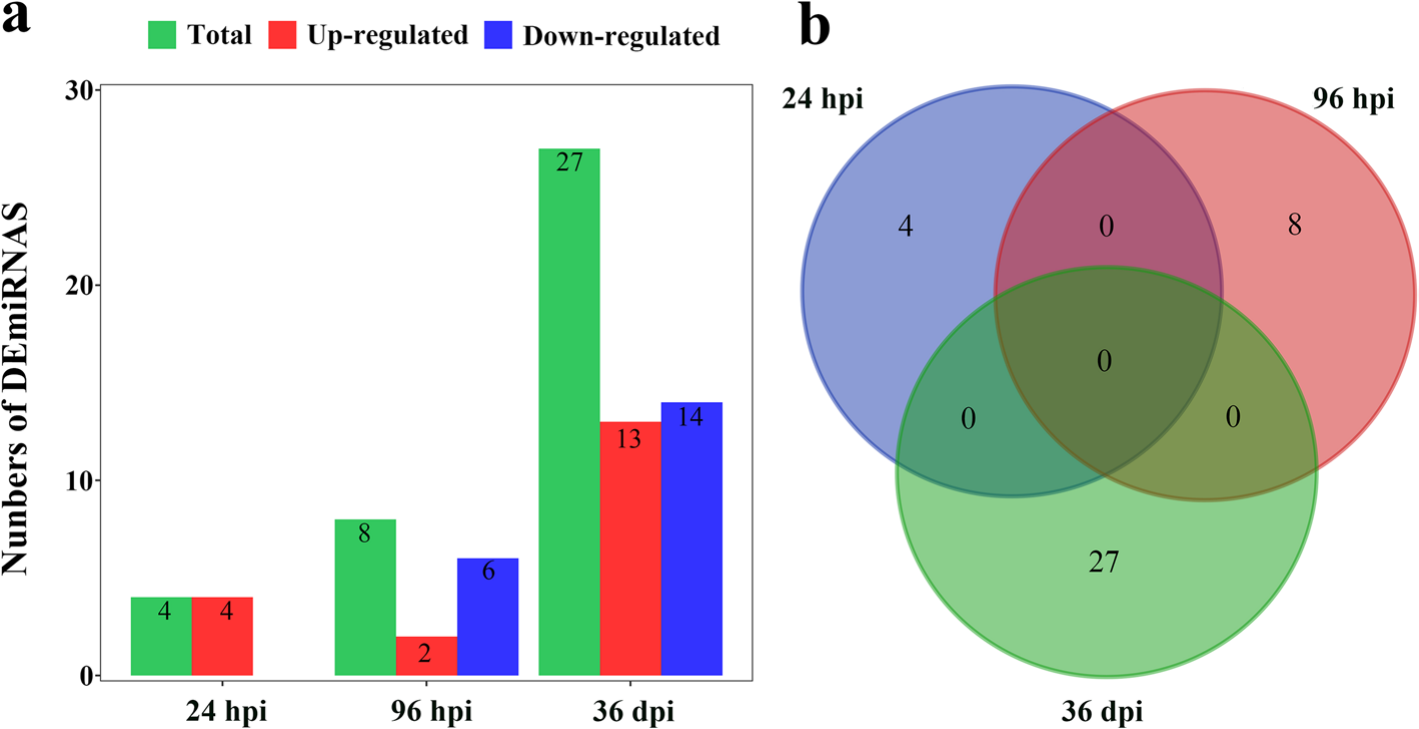
Comparison of differentially expressed (DE) miRNAs (DEmiRNAs) in the bone marrow of Beagle dogs infected by *Toxocara canis* at 24 h post-infection (hpi), 96 hpi, and 36 days post-infection (dpi). **a** The number of DEmiRNAs at 24 hpi, 96 hpi and 36 dpi. Green represents the total number of DEmiRNAs, red represents the number of up-regulated DEmiRNAs, and blue represents the number of down-regulated DEmiRNAs. **b** Venn diagram showing the numbers of common and unique DEmiRNAs at the three infection stages

**Fig. 2:**
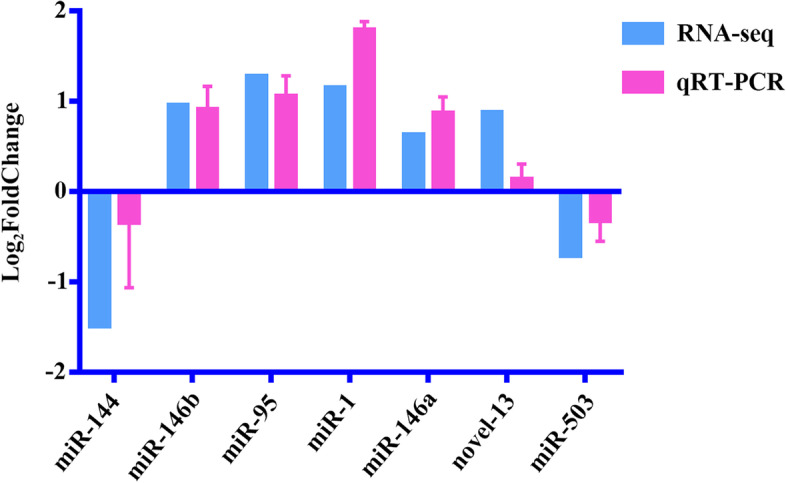
Validation of differentially expressed (DE) miRNAs (DEmiRNAs) using qRT-PCR. The error bar represents the standard deviation based on three repetitions. The X-axis shows DEmiRNAs, and the Y-axis shows the relative expression level of DEmiRNAs

A total of 39 DEmiRNAs were identified in this study. Among them, four DEmiRNAs were identified at 24 hpi and all were up-regulated; 8 DEmiRNAs were identified at 96 hpi, including two up-regulated miRNAs and six down-regulated miRNAs; 27 DEmiRNAs were identified at 36 dpi, including 13 up-regulated miRNAs and 14 down-regulated miRNAs (Fig. 1a and Table S1). However, no overlapping DEmiRNAs between either of the three infection stages were identified (see the Venn diagram in Fig. 1b). The qRT-PCR validation results are depicted in Fig. 2, which showed that the qRT-PCR results were consistent with the overall trend of RNA-seq expression levels, further demonstrating that the RNA-seq results are reliable.

### Target gene prediction and GO annotation analysis of DEmiRNAs

**Fig. 3:**
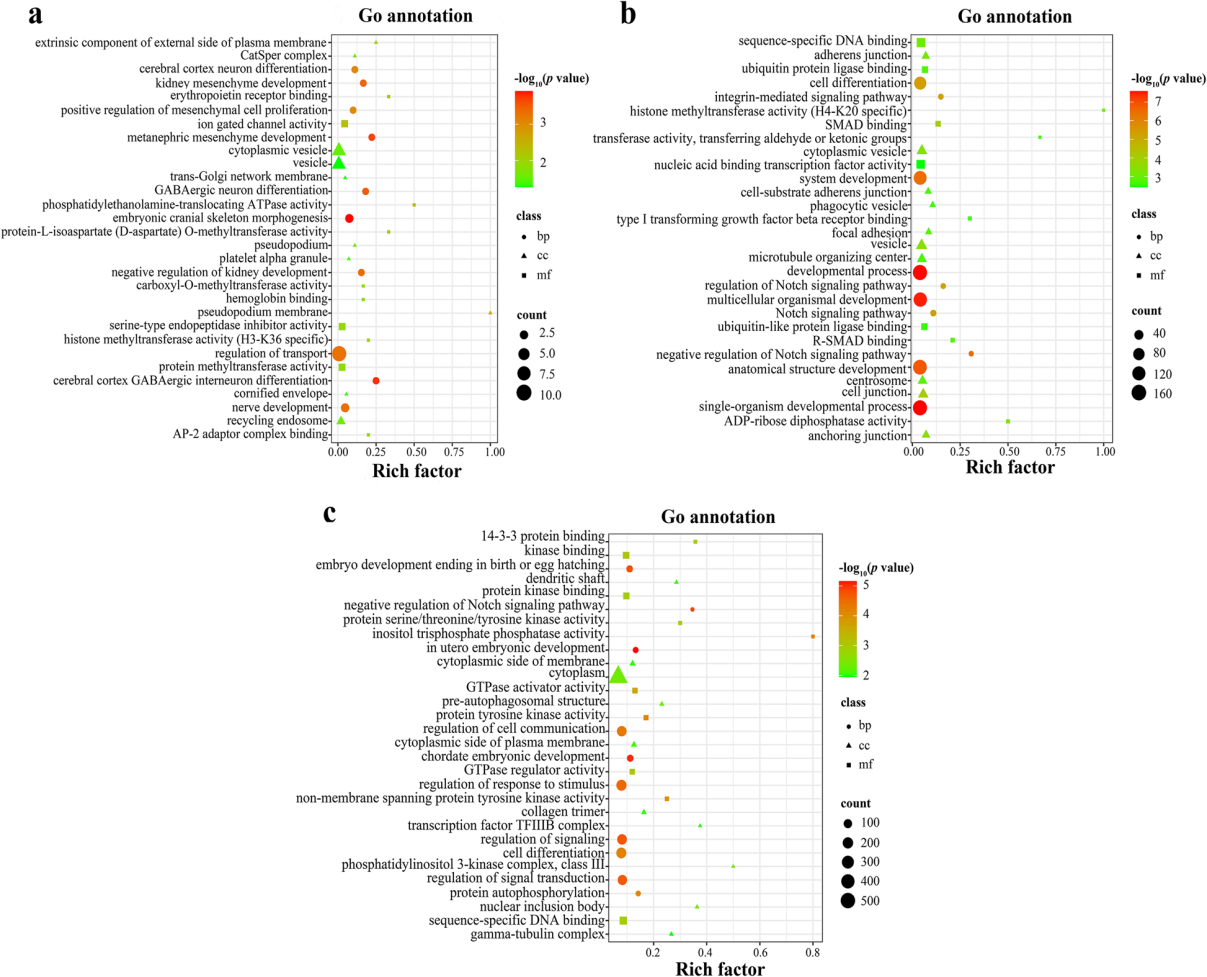
Scatter plots of the top 30 enriched Gene Ontology (GO) terms of differentially expressed (DE) miRNAs (DEmiRNAs) at (**a**) 24 h post-infection (hpi), (**b**) 96 hpi, and (**c**) 36 days post-infection (dpi). The X-axis represents the rich factor and the Y-axis shows the GO terms. The greater the rich factor, the greater the degree of term enrichment. The color of the dots indicates the enrichment score [-log_10_(*P* value)], where red indicates high enrichment, while green indicates low enrichment. The dot size indicates the number of the potential target gene of DEmiRNAs in the corresponding GO term. Larger dots indicate the greater number of potential target genes enriched in this GO term

A total of 48, 523, and 1,167 potential target genes of DEmiRNAs were predicted at 24 hpi, 96 hpi and 36 dpi, respectively (Table S2). GO annotation analysis revealed that a total of 31 potential target genes of two DEmiRNAs were significantly enriched in 384 GO terms at 24 hpi, with one immune-related GO term such as immune system development (GO: 0002520); a total of 476 potential target genes of 7 DEmiRNAs were significantly enriched in 836 GO terms at 96 hpi, with 26 immune-related GO term, such as cytokine production involved in immune response (GO:0002367), regulation of lymphocyte mediated immunity (GO:0002706), cell activation involved in immune response (GO:0002263); a total of 1,028 potential target genes of 17 DEmiRNAs were significantly enriched in 857 GO terms at 36 dpi, with 22 immune-related GO term, such as T-helper 17 type immune response (GO:0072538), neutrophil mediated immunity (GO:0002446), humoral immune response mediated by circulating immunoglobulin (GO:0002455) (Table S3). The top 10 significantly enriched biological process (BP), cellular component (CC), and molecular function (MF) terms of the three stages after *T. canis* infection are shown in Fig. 3.

### KEGG pathway enrichment analysis

**Fig. 4:**
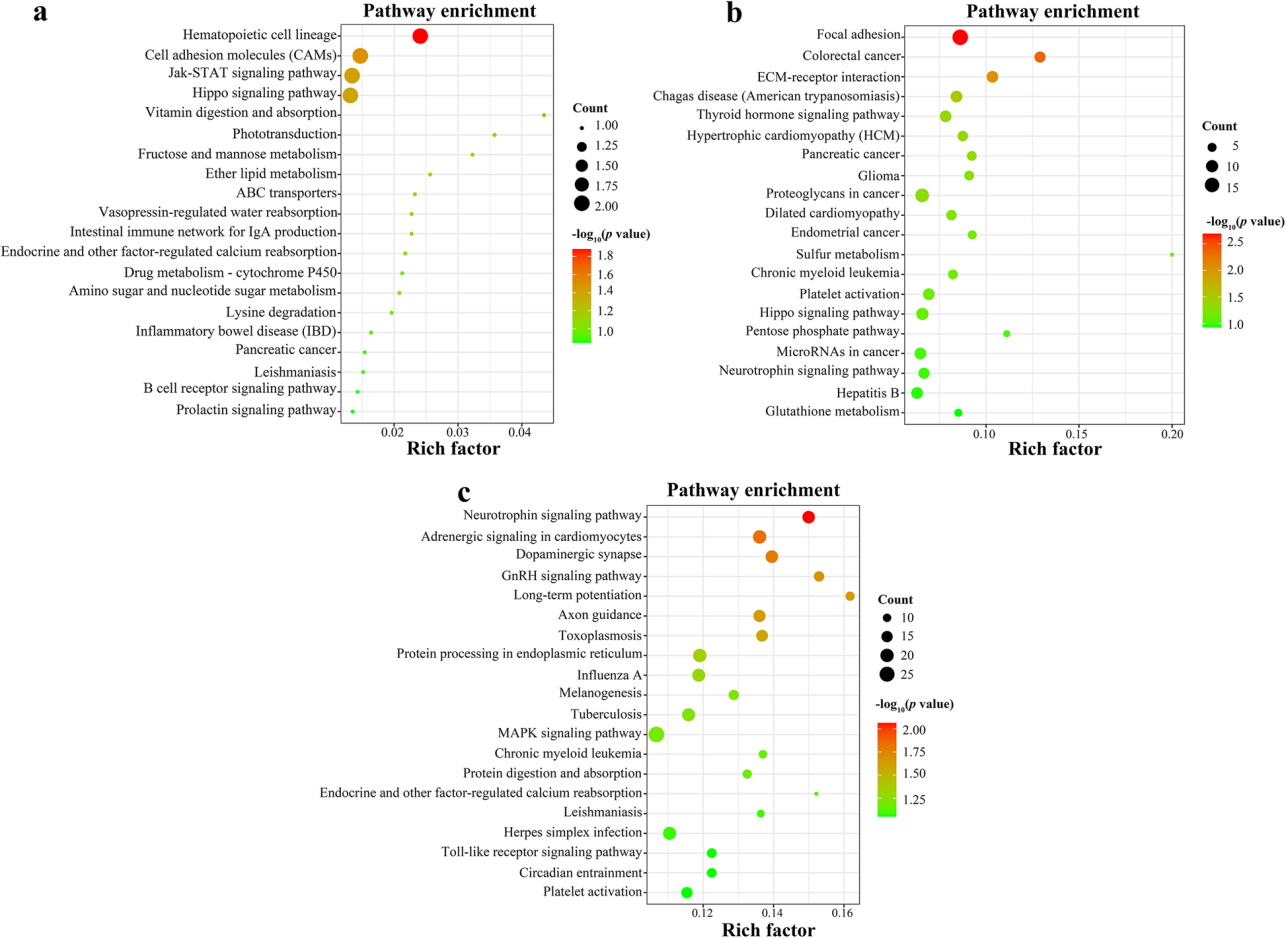
Scatter plots of the top 20 enriched Kyoto Encyclopedia of Genes and Genomes (KEGG) pathway of differentially expressed (DE) miRNAs (DEmiRNAs) at (**a**) 24 h post-infection (hpi), (**b**) 96 hpi, and (**c**) 36 days post-infection (dpi). The X-axis represents the rich factor; the Y-axis shows the KEGG pathways. The greater the rich factor, the greater the degree of pathways enrichment. The color of the dots indicates the enrichment score [-log_10_(*P* value)], where red indicates high enrichment, while green indicates low enrichment. The dot size indicates the number of the potential target gene of DEmiRNAs in the corresponding pathways. Larger dots indicate the greater number of potential target genes enriched in this pathway

KEGG pathway analysis revealed that seven potential target genes of two DEmiRNAs were significantly enriched in five signaling pathways at 24 hpi, such as Hematopoietic cell lineage (cfa04640), Cell adhesion molecules (CAMs) (cfa04514), Jak-STAT signaling pathway (cfa04630), Hippo signaling pathway (cfa04390), and Vitamin digestion and absorption (cfa04977); 35 potential target genes of 6 DEmiRNAs were significantly enriched in seven signaling pathways at 96 hpi, such as Focal adhesion (cfa04510), Colorectal cancer (cfa05210), ECM-receptor interaction (cfa04512), Chagas disease (American trypanosomiasis) (cfa05142), Thyroid hormone signaling pathway (cfa04919); a total of 83 potential target genes of 10 DEmiRNAs were significantly enriched in eight signaling pathways at 36 dpi, such as Neurotrophin signaling pathway (cfa04722), Adrenergic signaling in cardiomyocytes (cfa04261), Dopaminergic synapse (cfa04728), GnRH signaling pathway (cfa04912), Long-term potentiation (cfa04720) (Table S4). The top 20 representative pathways at the three infection stages are shown in Fig. 4.

## Discussion

Previous studies have shown that miRNAs can play critical roles in the pathogenesis of *T. canis* during the liver phase and lung phase of parasite development [16, 17]. However, the effect of *T. canis* infection on miRNA expression of dog bone marrow remains unclear. In this study, RNA-seq was used to examine the expression levels of miRNAs in bone marrow of Beagle dogs at different stages after *T. canis* infection, which provided useful resources for further understanding the pathogenesis of *T. canis* infection. 39 DEmiRNAs were identified during the three stages after *T. canis* infection, and most of them had multiple target genes, suggesting that these DEmiRNAs may exert a broader range of functions during *T. canis* infection in dogs.

We identified the least number of DEmiRNAs and potential target genes at the initial time point of infection and showed an increasing trend with the time of infection. At the same time, all DEmiRNAs identified at the early stage of infection were up-regulated, while a large proportion of DEmiRNAs was down-regulated at the later stage of infection. This is consistent with our anticipation of the stages of the host immune system response to infection. In the early stage of infection, a small number of DEmiRNAs in the host activates the innate immune response and prevents further invasion of *T. canis* through the inflammatory response. As the infection proceeds, at 96 hpi, the host activates the adaptive immune response through regulation of a portion of the dysregulated DEmiRNAs. At the late stage of infection, the host promotes the activation and differentiation of immune cells through many dysregulated DEmiRNAs. We speculate that this is closely related to the increasing complexity of parasite-host interactions.

### Target gene prediction and DEmiRNAs analysis

At 24 hpi, four DEmiRNAs, such as cfa-miR-122 and cfa-miR-193b, were predicted to be able to regulate 48 potential target genes. CD22 is a specific adhesion molecule on the surface of B lymphocytes, which can activate and regulate the function of B lymphocytes through ligand-dependent and non-dependent mechanisms, and can respond to exogenous antigen or self-antigen [18]. Recognition of antigens by the B-cell antigen receptor (BCR) is the main process in the B-cell immune response [19], and Toll-like receptors (TLRs) are the pathogen recognition receptor that initiates the innate immune response [20]. CD22 has been shown to inhibit BCR and TLR signaling in B cells [19, 21]. At 24 hpi, *T. canis* larvae hatch in the small intestine of the infected dogs, then reach the liver via the intestinal wall, lymphatic vessels and capillaries; and during this process, excretory-secretory products (ESPs) of *T. canis* larvae will induce the immune response of dogs [5]. *cd22* was predicted as a potential target gene for cfa-miR-193b in this study (Table S2). We speculate that up-regulated cfa-miR-193b could enhance the host immune response to *T. canis* infection, which could facilitate host resistance against *T. canis* infection. Erythropoietin (EPO) plays important role in the survival of erythroid progenitors by binding to its cell surface receptor, promoting cell survival, proliferation and differentiation in the production of mature RBCs [22]. In this study, *epo* could be down-regulated by up-regulated cfa-miR-122, which could affect the maturation of RBCs, and lead to anemia symptoms in puppies. The migration of *T. canis* larvae can cause damage to the body, resulting in a series of inflammatory reactions [5]. A previous study found that miR-382 contributes to promote the inflammatory responses in the host [23]. The up-regulated cfa-miR-382 induced by *T. canis* infection may be more specific on the expected effects of identified target genes in the pro-inflammatory response at the early infection stage in the infected dogs. In conclusion, the target genes predicted at 24 hpi can serve as host innate immune response components, promoting host resistance to *T. canis* infection, and also serving as a bridge that links innate and adaptive immunity.

At 96 hpi, eight DEmiRNAs, such as novel_328, were predicted to be able to regulate 523 potential target genes. The novel_328 was down-regulated at 96 hpi and was predicted to have multiple potential target genes in this study, such as transforming growth factor beta 1 (*tgfb1*) and thymocyte expressed, positive selection associated 1 (*tespa1*) (Table S2). TGFB1 is a pleiotropic and multifunctional growth factor that is mainly expressed in the immune system. It has potent immunomodulatory properties and can exert a potent anti-inflammatory function [24]. The down-regulation of novel_328 could contribute to the immune regulation and anti-inflammatory effect of the host by regulating the expression of *tgfb1*. TESPA1 is one of the members of TCR signaling, which is essential for T cell selection and maturation through regulating TCR signaling during T cell development [25]. *Tespa1*-deficient mice exhibit impaired thymocyte development, and the decrease of CD4^+^ and CD8^+^ T cells in the matured thymus [25]. The down-regulation of novel_328 in the puppy bone marrow may promote the puppy immune response to resist *T. canis* infection at 96 hpi by affecting the expression of *tespa1*. However, the biological function of *tgfb1* and *tespa1* in *T. canis* infection requires further experimental investigation. Therefore, we speculate that at 96 hpi, the activation of host adaptive immune response is closely associated with the potential target genes (*tgfb1* and *tespa1*), and the biological functions of *tgfb1* and *tespa1* in *T. canis* infection should be deeply investigated in subsequent studies.

At 36 dpi, 27 DEmiRNAs, such as cfa-miR-331 and novel_129, were predicted to be able to regulate 1,167 potential target genes. cfa-miR-331 and novel_129 were significantly down-regulated at 36 dpi. Semaphorin 7A (SEMA7A), acting as an effective immunomodulator, can be expressed in activated lymphocytes and myeloid cells, which can promote cell differentiation of the immune system [26], and can significantly increase Th1/Th17 cytokine secretion [27]. *Sema7a* was predicted to be a potential target gene of cfa-miR-331 in this study (Table S2). The down-regulation of cfa-miR-331 may promote host TH1 immune response in *T. canis* infection at 36 dpi by affecting the expression of *sema7a*. As a member of the IgSF, CD96 is involved in both immune and developmental processes [28]. A previous study showed that CD96 may contribute to the differentiation and activation of T cells, such as enhancing the activation and effector responses of CD8^+^ T cells [29]. *cd96* was predicted to be a potential target gene for novel_129 in this study (Table S2). The abnormal expression of novel_129 may be a self-protective manifestation in the host against *T. canis* infection through regulating the expression of *cd96.* In the late stages of *T. canis* infection in dogs, the predicted potential target genes act as immunomodulators, which may contribute to host self-protection and organismal stability.

### GO annotation analysis of DEmiRNAs

At 24 hpi, GO annotation analysis showed that 384 GO terms were significantly enriched; among which, 351, 24 and 9 GO terms were included in the three categories of BP, MF and CC, respectively (Table S3). EPO, a glycoprotein hormone, is a key cytokine regulator of erythropoiesis in the bone marrow and plays an important role in erythropoiesis by Epo/Epo receptor (EpoR) signaling pathways [30]. At 24 hpi, GO terms such as response to erythropoietin, cellular response to erythropoietin, erythropoietin-mediated signaling pathway and erythrocyte maturation were significantly enriched in the bone marrow of infected puppies,, which may be a compensatory response to visceral injury and anemia of the puppies caused by *T. canis* infection at the early stage. These results indicated that puppies may mobilize the production of RBCs at this time to promote the recovery of body damage.

At 96 hpi, 836 GO terms were significantly enriched; among which, 674, 95 and 67 GO terms were included in the three categories of BP, MF and CC, respectively (Table S3). ESPs of *T. canis* larvae can induce complex host immune responses during infection [8]. At 96 hpi, 476 potential target genes of seven DEmiRNAs were mainly associated with GO terms of BP; among which, many potential target genes were associated with immune reaction, such as T cell mediated immunity, regulation of T cell mediated immunity, regulation of humoral immune response, regulation of adaptive immune response and regulation of B cell mediated immunity. The significant enrichment of these immune response-related GO terms indicated that *T. canis* infection has caused a strong immune response in the infected puppies.

At 36 dpi, 857 GO terms were significantly enriched; among which 692, 112 and 53 GO terms were included in the three categories of BP, MF and CC, respectively (Table S3). MAP kinase, located in the protein kinase cascade, is the main component that controls embryogenesis, cell differentiation, cell proliferation and cell death pathways [31]. Mitogen-activated protein kinases (MAPKs) pathway can be activated by various pro-inflammatory factors or inflammatory reactions, which plays an important role in mediating effective inflammatory response and cytokine production [32, 33]. In this study, some potential target genes of DEmiRNAs were significantly enriched in positive regulation of MAPK cascade, regulation of MAP kinase activity, MAP kinase activity, stress-activated MAPK cascade and activation of MAPKKK activity. When *T. canis* returns to the intestine of the puppies, it will continue to cause inflammatory response, which may not be limited to the intestine and also occur in the bone marrow. Biological processes related to MAPKs could play important roles in the host at the late stage of *T. canis* infection.

### KEGG pathway enrichment analysis

At 24 hpi, Cell adhesion molecules (CAMs), Hematopoietic cell lineage, Vitamin digestion and absorption, Hippo signaling pathway, and Jak-STAT signaling pathway were significantly enriched (Fig. 4a). The CAMs of IgSF regulate some vital processes in the life course of cells, including cell proliferation and cell differentiation in time and space [34]. In the process of inflammation, CAMs can mediate the adhesion between leukocytes and endothelial cells, so that leukocytes can migrate to the inflammatory site [35]. A previous study found that CAMs play important roles in goats resistance to gastrointestinal nematodes (GINs) infection [36]. The CAMs may stimulate host immune function in the early stage of *T. canis* infection, which maintains the stability of the body and plays an important role in host defense against *T. canis* invasion.

At 96 hpi, the potential target genes of DEmiRNAs were significantly enriched in Focal adhesion and ECM-receptor interaction (Fig. 4b). ECM-receptor interaction affects cellular activities, including cell proliferation, differentiation, migration and survival [37]. ECM-receptor interaction as an immune-related pathway helps parasites adapt to the host [38]. We speculate that ECM-receptor interaction may contribute to the migration of *T. canis* larvae to suitable survival sites and avoid the clearance of the host immune system. The Focal adhesion pathway can perform a variety of biological functions, including the immune response and the barrier function, as well as promote the migration of neutrophils from blood vessels to infected sites by participating in endothelial conformational changes during the inflammatory process [39, 40]. A previous study has shown that gene expression related to focal adhesion may change in the host infected with *Trichinella spiralis*, leading to focal adhesion changes and the formation of epithelial monolayer tight junctions, which may be beneficial to host defense against *H. spiralis* infection [39]. Puppies could resist *T. canis* larvae infection or migration to other tissues by focal adhesion pathway at 96 hpi.

At 36 dpi, the target genes of DEmiRNAs were enriched in the Neurotrophin signaling pathway, Long-term potentiation, Axon guidance and Protein processing in endoplasmic reticulum (Fig. 4c). Neurotrophins and their receptors in neurotrophin signaling pathway are widely expressed in the immune system [41]. Neurotrophins can regulate the functions of lymphocytes and eosinophils, and promote B cell maturation, while also acting on hematopoietic lineage cells to regulate their hematopoietic function [42]. Neurotrophins are increased during the inflammatory response, parasitic infection, etc. [42]. We speculate that the Neurotrophin signaling pathway may play a critical role in host inflammation and tissue repair at 36 dpi by activating immune inflammatory cells through this pathway.

## Conclusions

The present study examined the miRNA expression profiles in the bone marrow of Beagle puppies during the different stages of *T. canis* infection. The findings reveal dynamic changes in expression profiles of miRNAs, reflecting the complexity of the *T. canis*-host interaction. cfa-miR-193b, novel_328, and cfa-miR-331 are associated with the host immune response. novel_129 could regulate host self-protection mechanisms. cfa-miR-382 may contribute to promote host inflammatory responses. GO and KEGG pathway analysis revealed that these DEmiRNAs were involved in many critical biological processes and the regulation of diseases and immune signaling pathways. These findings also provided valuable information for further understanding the pathogenesis of *T. canis* infection.

## Materials and methods

### The collection of bone marrow tissue samples

A total of 18 Beagle dogs from 3 litters, 6–7 weeks old, were purchased from and housed at the National Canine Laboratory Animal Resource Center (Guangzhou, China). All puppies were examined using the sugar flotation method to ensure that there was no intestinal parasite infection. Moreover, each puppy’s serum was tested by indirect ELISA using larval excretory-secretory antigen to eliminate the *T. canis* infection [10]. According to the migration time points of the *T. canis* in definitive dog host, these puppies were divided into three time point groups: 24 h post-infection (hpi), 96 hpi, and 36 days post-infection (dpi). Each time point group included infected dogs and uninfected control dogs. Puppies from the same litter are equally distributed to reduce the influence of individual factors. Infected puppies orally received 1 mL of normal saline solution containing 300 infectious *T. canis* eggs, while uninfected control puppies received 1 mL of normal saline orally. At each specified time point post-infection as described above, puppies were anesthetized using Zoletil 50™ (Virbac, Nice, France), and then were euthanized through injecting KCl solution into the heart [10]. Then, the tibial bone marrow was rapidly separated aseptically and was stored in liquid nitrogen for RNA extraction. The potential infectious waste materials, such as puppy feces, infectious eggs and discarded tissues, were autoclaved before disposal.

### RNA isolation

Total RNA was extracted from bone marrow samples with Trizol (Life Technologies, Carlsbad, USA). The genomic DNA in extracted RNA was removed with DNase I (NEB, Ipswich, USA). The purity and concentration of above RNA were determined with a Qubit® 2.0 Flurometer (Life Technologies, Carlsbad, USA). The integrity of the isolated RNA was verified using an Agilent Bioanalyzer 2100 system (Agilent Technologies, SantaClara, USA). The high-quality RNAs with RNA integrity number (RIN) value > 8.0 were used to construct the small RNA-seq libraries.

### Library preparation and small RNA sequencing

Three μg total RNA per sample of puppy bone marrow was used as input material to generate the small RNA-seq library by using NEBNext® Multiplex Small RNA Library Prep Set for Illumina® (NEB, Ipswich, USA), and the index codes of 3' SR Adaptor (NEB, Ipswich, USA) were added to attribute sequences to each puppy bone marrow sample, following the manufacturer’s recommendations. Then, first-strand cDNA was synthesized, and the cDNA was amplified using LongAmp Taq 2X Master Mix, SR Primer for Illumina and index (X) primer. The PCR products were electrophoresed by 8% PAGE (100 V, 80 min), and the DNA fragments (including aptamers) of approximately 140–160 bp were recovered and purified to assess the quality on the Agilent Bioanalyzer 2100 system using DNAHigh Sensitivity Chips. The clustering of the index-coded samples was performed on a cBot Cluster Generation System using TruSeq SR Cluster Kit v3-cBot-HS (Illumia, San Diego, USA) according to the manufacturer’s instructions. After cluster generation, the libraries were sequenced on an Illumina Hiseq 2500 platform and 50 bp long single-end reads were generated.

### Identification of miRNAs

Raw data (raw reads) that containing poly-N, with 5' adapter contaminants, without 3' adapter or the insert tag and low-quality reads were firstly filtered by custom perl and python scripts to obtain clean data (clean reads). Then, the Q20, Q30 and GC-content of raw data were calculated. The small RNAs of clean reads ranging from 18–30 nt were mapped to reference sequences by Bowtie to search known miRNAs [43]. Using the known miRNAs of *Canis familiaris* in MiRbase 22.0 as a reference, the improved software miRdeep 2 and srna-tools-cli were used to identify potential miRNAs and draw the stem-loop structure of the identified miRNAs [44, 45]. According to the characteristics of the hairpin structure of miRNA precursor, miREvo and miRdeep 2 were used to predict the novel miRNAs, and the secondary structure and minimum free energy of the small RNA tags that were unannotated in the preceding steps were analyzed [44, 46]. To ensure that each unique small RNA was mapped to only one annotation, the following priority rule was set up: known miRNA > rRNA > tRNA > snRNA > snoRNA > NAT-siRNA > novel miRNA > ta-siRNA.

### The prediction of miRNA target genes and the enrichment analysis of GO and KEGG

The prediction of miRNA target genes was conducted by miRanda [47]. The expression level of miRNA was estimated by transcript per million (TPM) [48]. The transcriptional level of miRNA was assessed by the DESeq R package, and the DEmiRNA was identified by the thresholds of *P* values < 0.05 between infected dogs and uninfected control dogs [49]. GO annotation and KEGG pathway analysis of the target genes of DEmiRNAs were performed by GOseq R package and KOBAS 3.0, respectively [50–52]. *P* values < 0.05 were considered significantly enriched for GO and KEGG terms.

### The qRT-PCR validation of RNA-Seq results

To validate the accuracy of the RNA-seq data, seven DEmiRNAs identified at 36 dpi were selected for verification by quantitative real-time PCR (qRT-PCR). The qRT-PCR quantitative experiment was performed on LightCycler480 (Roche, Basle, Switzerland). The miRNA reverse transcription step, qRT-PCR amplification procedures and melting curve analysis were performed strictly as previously described [17]. The selected DEmiRNAs and their PCR primers are summarized in Table S5. The verification results were calculated by the 2^−ΔΔCT^ method, with U6 small ribonucleic acid as the internal reference for normalizing the level of miRNAs.

## Supplementary Information

**Additional file 1: Table S1.** The differently expressed miRNAs (DEmiRNAs) (*P* < 0.05) in puppy bone marrow at different infection stages. **Table S2.** The potential target genes of the differentially expressed miRNAs (DEmiRNAs) at different infection stages. **Table S3.** The differential enriched Gene Ontology (GO) terms of potential target genes of the differentially expressed miRNAs. **Table S4.** The KEGG pathways of the differentially expressed miRNAs (DEmiRNAs) at different infection stages. **Table S5.** The primers used in the qRT-PCR experiment.

## Data Availability

The datasets supporting the findings of this article are included within the paper and its supplementary materials. The datasets generated during the current study are available in the NCBI Short Read Archive repository under the BioProject number PRJNA843610.

## Abbreviations

LncRNA: Long non-coding RNA
DE: Differentially expressed
hpi: Hours post-infection
dpi: Days post-infection
GO: Gene Ontology
KEGG: Kyoto Encyclopedia of Genes and Genomes
qRT-PCR: Quantitative Real-time PCR
RNA-seq: RNA-sequencing

## References

[1] Ma G, Holland CV, Wang T, Hofmann A, Fan CK, Maizels RM (2018). Human toxocariasis. Lancet Infect Dis.

[2] Rostami A, Riahi SM, Holland CV, Taghipour A, Khalili-Fomeshi M, Fakhri Y (2019). Seroprevalence estimates for toxocariasis in people worldwide: a systematic review and meta-analysis. PLoS Negl Trop Dis.

[3] Ma G, Rostami A, Wang T, Hofmann A, Hotez PJ, Gasser RB (2020). Global and regional seroprevalence estimates for human toxocariasis: a call for action. Adv Parasitol.

[4] Rostami A, Riahi SM, Hofmann A, Ma G, Wang T, Behniafar H (2020). Global prevalence of *Toxocara* infection in dogs. Adv Parasitol.

[5] Schnieder T, Laabs EM, Welz C (2011). Larval development of *Toxocara canis* in dogs. Vet Parasitol.

[6] El-Naccache DW, Chen F, Chen N, Gause WC (2020). The net effect of neutrophils during helminth infection. Cell Host Microbe.

[7] Shamri R, Xenakis JJ, Spencer LA (2011). Eosinophils in innate immunity: an evolving story. Cell Tissue Res.

[8] Zheng WB, Zou Y, Zhu XQ, Liu GH (2020). *Toxocara* "omics" and the promises it holds for medicine and veterinary medicine. Adv Parasitol.

[9] Wangchuk P, Lavers O, Wishart DS, Loukas A (2020). Excretory/secretory metabolome of the zoonotic roundworm parasite *Toxocara canis*. Biomolecules.

[10] Zheng WB, Zou Y, Liu Q, Hu MH, Elsheikha HM, Zhu XQ (2021). *Toxocara canis* infection alters lncRNA and mRNA expression profiles of dog bone marrow. Front Cell Dev Biol.

[11] Zheng WB, Zou Y, He JJ, Liu GH, Hu MH, Zhu XQ (2021). Proteomic alterations in the plasma of beagle dogs induced by *Toxocara canis* infection. J Proteomics.

[12] Liu Q, Tuo W, Gao H, Zhu XQ (2010). MicroRNAs of parasites: current status and future perspectives. Parasitol Res.

[13] Saliminejad K, Khorram Khorshid HR, Soleymani Fard S, Ghaffari SH (2019). An overview of microRNAs: biology, functions, therapeutics, and analysis methods. J Cell Physiol.

[14] Alizadeh Z, Mahami-Oskouei M, Spotin A, Ahmadpour E, Cai P, Sandoghchian Shotorbani S (2022). MicroRNAs in helminth parasites: a systematic review. Curr Mol Med.

[15] Ma G, Luo Y, Zhu H, Luo Y, Korhonen PK, Young ND (2016). MicroRNAs of *Toxocara canis* and their predicted functional roles. Parasit Vectors.

[16] Zou Y, Zheng WB, He JJ, Elsheikha HM, Zhu XQ, Lu YX (2020). *Toxocara canis* differentially affects hepatic microRNA expression in beagle dogs at different stages of infection. Front Vet Sci.

[17] Zheng WB, Zou Y, He JJ, Elsheikha HM, Liu GH, Hu MH (2021). Global profiling of lncRNAs-miRNAs-mRNAs reveals differential expression of coding genes and non-coding RNAs in the lung of beagle dogs at different stages of *Toxocara canis* infection. Int J Parasitol.

[18] Poe JC, Fujimoto Y, Hasegawa M, Haas KM, Miller AS, Sanford IG (2004). CD22 regulates B lymphocyte function in vivo through both ligand-dependent and ligand-independent mechanisms. Nat Immunol.

[19] Jellusova J, Nitschke L (2011). Regulation of B cell functions by the sialic acid-binding receptors siglec-G and CD22. Front Immunol.

[20] Aloor JJ, Azzam KM, Guardiola JJ, Gowdy KM, Madenspacher JH, Gabor KA (2019). Leucine-rich repeats and calponin homology containing 4 (Lrch4) regulates the innate immune response. J Biol Chem.

[21] Kawasaki N, Rademacher C, Paulson JC (2011). CD22 regulates adaptive and innate immune responses of B cells. J Innate Immun.

[22] Suresh S, Rajvanshi PK, Noguchi CT (2019). The many facets of erythropoietin physiologic and metabolic response. Front Physiol.

[23] Wang X, Xue N, Zhao S, Shi Y, Ding X, Fang Y (2020). Upregulation of miR-382 contributes to renal fibrosis secondary to aristolochic acid-induced kidney injury via PTEN signaling pathway. Cell Death Dis.

[24] Yang YC, Zhang N, Van Crombruggen K, Hu GH, Hong SL, Bachert C (2012). Transforming growth factor-beta1 in inflammatory airway disease: a key for understanding inflammation and remodeling. Allergy.

[25] Wang D, Zheng M, Lei L, Ji J, Yao Y, Qiu Y (2012). Tespa1 is involved in late thymocyte development through the regulation of TCR-mediated signaling. Nat Immunol.

[26] Ghofrani J, Lucar O, Dugan H, Reeves RK, Jost S (2019). Semaphorin 7A modulates cytokine-induced memory-like responses by human natural killer cells. Eur J Immunol.

[27] Xie J, Wang H (2017). Semaphorin 7A as a potential immune regulator and promising therapeutic target in rheumatoid arthritis. Arthritis Res Ther.

[28] Gong J, Zhu C, Zhuang R, Song C, Li Q, Xu Z (2009). Establishment of an enzyme-linked immunosorbent assay system for determining soluble CD96 and its application in the measurement of sCD96 in patients with viral hepatitis B and hepatic cirrhosis. Clin Exp Immunol.

[29] Chiang EY, de Almeida PE, de Almeida Nagata DE, Bowles KH, Du X, Chitre AS (2020). CD96 functions as a co-stimulatory receptor to enhance CD8^+^ T cell activation and effector responses. Eur J Immunol.

[30] Kim J, Jung Y, Sun H, Joseph J, Mishra A, Shiozawa Y (2012). Erythropoietin mediated bone formation is regulated by mTOR signaling. J Cell Biochem.

[31] Pearson G, Robinson F, Beers Gibson T, Xu BE, Karandikar M, Berman K (2001). Mitogen-activated protein (MAP) kinase pathways: regulation and physiological functions. Endocr Rev.

[32] Yong HY, Koh MS, Moon A (2009). The p38 MAPK inhibitors for the treatment of inflammatory diseases and cancer. Expert Opin Investig Drugs.

[33] Wang Y, Dai X, Liu Y, Li J, Liu Z, Yin P (2016). MTUS1 silencing promotes *E*-selectin production through p38 MAPK -dependent CREB ubiquitination in endothelial cells. J Mol Cell Cardiol.

[34] Steinbacher T, Kummer D, Ebnet K (2018). Junctional adhesion molecule-a: functional diversity through molecular promiscuity. Cell Mol Life Sci.

[35] Ulbrich H, Eriksson EE, Lindbom L (2003). Leukocyte and endothelial cell adhesion molecules as targets for therapeutic interventions in inflammatory disease. Trends Pharmacol Sci.

[36] Bhuiyan AA, Li J, Wu Z, Ni P, Adetula AA, Wang H (2017). Exploring the genetic resistance to gastrointestinal nematodes infection in goat using RNA-sequencing. Int J Mol Sci.

[37] Jones FS, Jones PL (2000). The tenascin family of ECM glycoproteins: structure, function, and regulation during embryonic development and tissue remodeling. Dev Dyn.

[38] Li M, Huang Q, Wang J, Li C (2018). Differential expression of microRNAs in *Portunus trituberculatus* in response to *Hematodinium* parasites. Fish Shellfish Immunol.

[39] Ma XH, Ren HJ, Peng RY, Li Y, Ming L (2020). Comparative expression profiles of host circulating miRNAs in response to *Trichinella spiralis* infection. Vet Res.

[40] Parsons SA, Sharma R, Roccamatisi DL, Zhang H, Petri B, Kubes P (2012). Endothelial paxillin and focal adhesion kinase (FAK) play a critical role in neutrophil transmigration. Eur J Immunol.

[41] Vega JA, García-Suárez O, Hannestad J, Pérez-Pérez M, Germanà A (2003). Neurotrophins and the immune system. J Anat.

[42] Aloe L, Simone MD, Properzi F (1999). Nerve growth factor: a neurotrophin with activity on cells of the immune system. Microsc Res Tech.

[43] Langmead B, Trapnell C, Pop M, Salzberg SL (2009). Ultrafast and memory-efficient alignment of short DNA sequences to the human genome. Genome Biol.

[44] Friedländer MR, Mackowiak SD, Li N, Chen W, Rajewsky N (2012). miRDeep2 accurately identifies known and hundreds of novel microRNA genes in seven animal clades. Nucleic Acids Res.

[45] Moxon S, Schwach F, Dalmay T, Maclean D, Studholme DJ, Moulton V (2008). A toolkit for analysing large-scale plant small RNA datasets. Bioinformatics.

[46] Wen M, Shen Y, Shi S, Tang T (2012). miREvo: an integrative microRNA evolutionary analysis platform for next-generation sequencing experiments. BMC Bioinformatics.

[47] Enright AJ, John B, Gaul U, Tuschl T, Sander C, Marks DS (2003). MicroRNA targets in *Drosophila*. Genome Biol.

[48] Zhou L, Chen J, Li Z, Li X, Hu X, Huang Y (2010). Integrated profiling of microRNAs and mRNAs: microRNAs located on Xq27.3 associate with clear cell renal cell carcinoma. PLoS One.

[49] Love MI, Huber W, Anders S (2014). Moderated estimation of fold change and dispersion for RNA-seq data with deseq2. Genome Biol.

[50] Young MD, Wakefield MJ, Smyth GK, Oshlack A (2010). Gene ontology analysis for RNA-seq: accounting for selection bias. Genome Biol.

[51] Xie C, Mao X, Huang J, Ding Y, Wu J, Dong S (2011). KOBAS 2.0: A web server for annotation and identification of enriched pathways and diseases. Nucleic Acids Res.

[52] Kanehisa M, Furumichi M, Sato Y, Ishiguro-Watanabe M, Tanabe M (2021). KEGG: integrating viruses and cellular organisms. Nucleic Acids Res.

