# Supplementary material for: Differential miRNA expression profiles in the bone marrow of Beagle dogs at different stages of Toxocara canis infection
Source: BMC Genomics. 2022 Dec 22;23:847. doi: 10.1186/s12864-022-09081-8 (PMC9773451; doi:10.1186/s12864-022-09081-8)
Supplement: Supplementary file 1 — Additional file 1: Table S1. The differently expressed miRNAs (DEmiRNAs) (P < 0.05) in puppy bone marrow at different infection stages. Table S2. The potential target genes of the differentially expressed miRNAs (DEmiRNAs) at different infection stages. Table S3. The differential enriched Gene Ontology (GO) terms of potential target genes of the differentially expressed miRNAs. Table S4. The KEGG pathways of the differentially expressed miRNAs (DEmiRNAs) at different infection stages. Table S5. The primers used in the qRT-PCR experiment. [file 12864_2022_9081_MOESM1_ESM.zip › Additional file 1-updated/Table S5.docx]

**Table S5.** The primers used in the qRT-PCR experiment.

| **miRNAs** | **Primer** | **Sequence (5′ to 3′)** |
| --- | --- | --- |
| U6^*^ | Forward primer | CGCTTCGGCAGCACATATAC |
| cfa-miR-144 | Forward primer | UACAGUAUAGAUGAUGUACUAG |
| cfa-miR-146b | Forward primer | UGAGAACUGAAUUCCAUAGGCU |
| cfa-miR-95 | Forward primer | UUCAACGGGUAUUUAUUGAGCA |
| cfa-miR-1 | Forward primer | UGGAAUGUAAAGAAGUAUGUA |
| cfa-miR-146a | Forward primer | UGAGAACUGAAUUCCAUGGGUU |
| novel-13 | Forward primer | CGCGACCUCAGAUCAGACGG |
| cfa-miR-503 | Forward primer | UAGCAGCGGGAACAGUACUG |

^*^The U6 small nuclear RNA (snRNA) was used as an internal control gene.
